# Effect of kinesio taping on low back pain during pregnancy: a systematic review and meta-analysis

**DOI:** 10.1186/s12884-021-04197-3

**Published:** 2021-10-25

**Authors:** Xiali Xue, Yan Chen, Xiaorong Mao, Huan Tu, Xinwei Yang, Zhongyi Deng, Ning Li

**Affiliations:** 1 Institute of Sports Medicine and Health, Chengdu Sport University, 2 Tiyuan Road, Wuhou District, Chengdu, 610041 Sichuan China; 2 Department of Gynecology and Obstetrics, Chengdu First People’s Hospital, Chengdu, 610095 Sichuan China; 3 Sichuan Academy of Medical Sciences and Sichuan People’s Hospital, Chengdu, 610072 Sichuan China; 4 School of Sports Medicine and Health, Chengdu Sport University, Chengdu, 610041 Sichuan China

**Keywords:** Kinesio taping, Pregnancy, Low back pain, Meta-analysis, Systematic review

## Abstract

**Background:**

Low back pain during pregnancy affects the normal daily activities of pregnant women to a certain extent. Current studies have shown that Kinesio Taping (KT) may be a treatment for low back pain during pregnancy, but there is still a lack of key evidence. The purpose of this study is to evaluate the efficacy and safety of KT in the treatment of low back pain during pregnancy.

**Methods:**

PubMed, Web of Science, The Cochrane Library, Scopus, Embase, Wanfang Data, CNKI, and VIP databases were searched to collect randomized controlled trials (RCTs) of the efficacy of KT intervention on low back pain in women during pregnancy. The retrieval time limit is from the establishment of the database to April 2021. Two researchers independently screened the literature, extracted the data, and evaluated the risk of bias in the included studies. Meta-analysis was performed using RevMan5.3 software.

**Results:**

A total of 7 RCTs were included, including 444 patients. Meta-analysis results showed that: Compared with the control group, KT intervention could significantly improve low back pain and dysfunction, VAS score (− 1.62, 95%CI − 2.08 to − 1.16, *P* < 0.00001, I^2^ = 77%), RMDQ score (− 1.00, 95%CI − 1.54 to − 0.46, *P* = 0.0003, I^2^ = 80%); The results of the meta-analysis of the subgroup showed that compared with the control group, the KT intervention was less than or equal to 1 week, and the waist pain and dysfunction were improved, with statistically significant differences. The difference in the improvement of low back pain was statistically significant after KT intervention for more than 1 week, but there was no statistically significant difference in RMDQ score (− 1.25, 95%CI − 2.66 to 0.15, *P* = 0.08, I^2^ =77%). Compared with the control group, KT intervention improved low back pain in the second and third trimesters, and the difference was statistically significant.

**Conclusion:**

KT has a positive effect on the improvement of low back pain during pregnancy, KT intervention can significantly improve pregnant women’s low back pain and dysfunction problems, improve the quality of life. It is suggested that future research should focus on the prevention and treatment of low back pain during pregnancy to provide more research data for improving women’s health.

**Supplementary Information:**

The online version contains supplementary material available at 10.1186/s12884-021-04197-3.

## Introduction

Pregnancy-related low back pain (PLBP) is a common problem of pregnant women during pregnancy. It is a physiological pathology that only appears during pregnancy and postpartum. The etiology is not clear, but the main potential factors include hormones, biomechanics, post-traumatic or degenerative diseases, pre-pregnancy low back pain history, and psychosocial factors [1]. The prevalence of low back pain during pregnancy has been reported to range from 20 to 90%, with most studies reporting a prevalence greater than 50% [2–4]. Pain becomes very serious with the progress of pregnancy, which has a great impact on the daily life and sleep of pregnant women, and seriously reduces their quality of life [5–8], It also increases the risk of postpartum anxiety and depression [9]. Studies have reported that women who suffer from severe low back pain during pregnancy are at an extremely high risk of developing new severe low back pain during subsequent pregnancy and later in life [10]. Therefore, the problem of low back pain during pregnancy can’t be ignored, and timely intervention is needed to improve. Currently, physiotherapy is used mainly for the treatment of low back pain during pregnancy due to the inexperience of clinicians regarding treatment options and concerns that other treatments may have harmful effects on fetal development.

Kinesio Taping (KT) is a non-invasive therapeutic technique developed by Dr. Kenzo Kase in 1973 [11]. Applied to the patient’s skin under tension in an elastic braid manner, it can be lengthways extended to 140% of its original length to treat a variety of musculoskeletal problems, such as injuries, pain, dysfunction, and other conditions, without limiting joint mobility [12, 13]. KT has been reported to correct joint dislocations, provide muscle support, activate endogenous pain relief systems, and eliminate tissue congestion [14, 15]. With the continuous innovation and progress of rehabilitation methods, KT has been applied to improve low back pain during pregnancy. Some clinical studies have found that KT can reduce pregnancy-related low back pain [16, 17]. However, other studies showed no significant difference in the improvement of low back pain between the control group and the KT [18, 19]. Therefore, this study conducted a comprehensive evaluation of relevant research results at home and abroad using meta-analysis, to provide an evidence-based basis for the effect of KT on low back pain during pregnancy.

## Methods

### Protocol and guidance

This study was performed by Preferred Reporting Items for Systematic Reviews and Meta-Analysis (PRISMA) [20]. The protocol of this review was published in PROSPERO (CRD42021249205).

#### Search strategy

We will search randomized controlled trials (RCTs) assessing the effect of KT on pregnancy with Low back pain, up to April 2021. The systematic literature search included the following databases: PubMed, Web of Science, The Cochrane Library, Scopus, Embase, Physiotherapy Evidence Database (PEDro), Chinese Biomedical Literature Database (CBM), Chinese National Knowledge Infrastructure Database (CNKI), Chinese Science, Wanfang Data, and the VIP databases. Search terms were a combination of keywords and free-text terms, the keywords and search strategy include (Taping or Elastic Taping or Kinesio Taping) AND (Pregnancy OR Pregnancies OR Gestation) AND Low back pain. All analyses were performed based on previously published studies; thus, no ethical approval or patient consent was required. To maximize the search for relevant articles, we reviewed reference lists of identified trials and systematic reviews. Take PubMed as an example, the specific retrieval strategy is shown in S1 File.

### Search strategy for the PubMed database

#### Eligibility and exclusion criteria

The inclusion criteria for screening eligible studies used the PICOS (Participants, Intervention, Comparator, Outcome, and Study design) model to select studies for this review.

1. Participants: patients with low back pain during pregnancy;
2. Intervention: patients received KT;
3. Comparator: patients received other treatment;
4. Outcomes: low back pain function and lumbar function improvement score;
5. Study design: Randomized clinical trial.

The exclusion criteria were as follows: studies were non-randomized controlled trials, non-peer-reviewed publications, case reports, case series, observational studies, opinion articles, with no language restrictions. Two researchers performed the search process and screened the articles according to the criteria independently. Disagreements between reviewers at each stage were resolved by discussion to reach a consensus.

#### Outcome types

The primary outcome was an improvement of pain (Visual Analogue Scale, VAS); The Visual Analogue Scale is a popular tool for the measurement of low back pain. It uses a ruler about 10 cm long, one side is marked with 10 scales, the two ends are “0” and “10” respectively, 0 indicates no pain, 10 indicates the most unbearable pain. The Secondary outcome was an improvement of lumbar function (Roland-Morris Dysfunction Questionnaire, RMDQ). The Roan-Morris Dysfunction Questionnaire can evaluate the short-term changes of low back pain before and after treatment, and it is a specific scoring scale for the evaluation of lumbar dysfunction. The questionnaire consisted of 24 questions closely related to low back pain. For each question, “yes” earned 1 point, and “no” earned 0 point. The highest score is 24 and the lowest is 0. The higher the score, the more serious the degree of lumbar dysfunction.

#### Study selection

The retrieved studies will be imported into Endnote X8 to remove duplicates. Two researchers (XXL and CY) will independently screen the titles and abstracts according to the pre-established inclusion and exclusion criteria. After that, the full text will be screened as a second filtration. Two researchers will crosscheck the included studies, and the third researcher (LN) will be involved if disagreements occur.

#### Data extraction

Article selection and data extraction were completed independently by two reviewers (DZY and TH), and a consensus was achieved by discussion. The following data were extracted from each included study: name of the first author, year of publication, study characteristics (sample size, interventions, treatment frequency, outcomes measure, and follow-up time), and participants’ characteristics (mean age, sex, and duration of disease). If the original data was unclear or lacking, the corresponding author was contacted to obtain further information.

#### Methodological quality assessment

This study used the PEDro tool (http://www.pedro.fhs.usyd.edu.au/scale_item.html) to assess the methodological quality of individual RCTs. Studies with PEDro scores ranging from 9 to 10 were considered methodologically to be of “excellent” quality. Studies with PEDro scores ranging from 6 to 8 were considered to be of “good” quality, while studies scoring between 4 to 5 were of “fair” quality. Studies that scored below 4 were of “poor” quality [21]. In this study, we considered a study awarded ≥6 points on the PEDro scale a high-quality study.

#### Data synthesis

Meta-analysis was performed with RevMan 5.3 software. Standard Mean Difference (SMD) data with its 95% Confidence Intervals (CI) was used as a measure of effect sizes to pool the results from each included study. Heterogeneity and inconsistencies among the included studies were assessed by chi square test and *I*^*2*^ statistics respectively. Results were graphically represented by a forest plot. When *P* > 0.10 or *I*^*2*^ ≤ 50%, the results of the associated studies were said to be homogenous or to have acceptable heterogeneity, and a fixed-effects model was utilized. When *P* ≤ 0.10 or *I*^*2*^ > 50%, it was considered that there was heterogeneity in the results of the multiple included studies, and subgroup analysis or sensitivity analysis was performed to identify the sources of heterogeneity. Then, the selected studies were removed one by one, and the overall correlation results and *I*^*2*^ were recalculated. A random-effects model was selected if the heterogeneity could not be eliminated [22]. The level of the meta-analysis was set as *P* < 0.05. Significance clinical heterogeneity was treated by subgroup analysis or sensitivity analysis, or by descriptive analysis only [23].

## Results

### Eligible studies

**Fig. 1:**
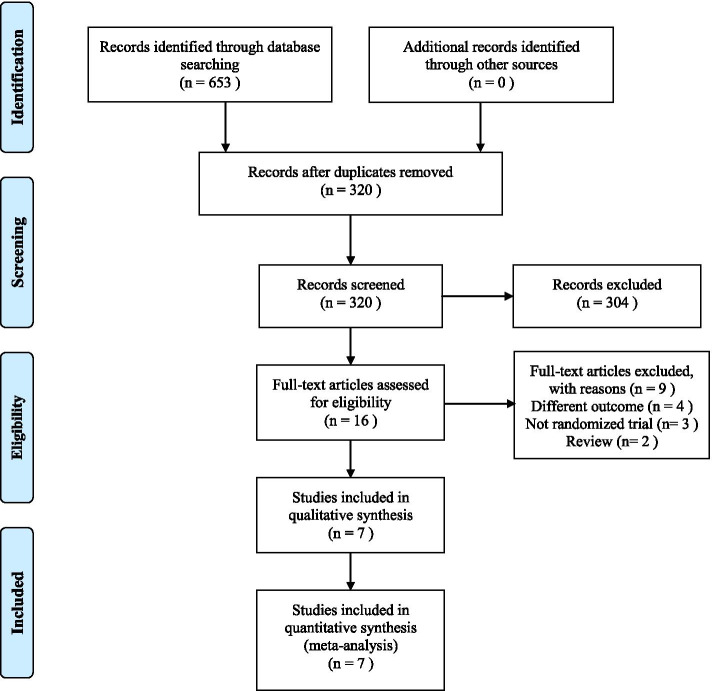
Flow diagram of the study selection process

A total of 653 articles were retrieved using the preliminary search strategy. After duplicates removing, 320 articles remained for review. A total of 304 articles were excluded because the data are incomplete. The remaining 16 full-text articles were evaluated; of these, Finally, 7 articles were included in the present study, the included studies were reported in English (four trials) and Chinese (three trials) [24–30] (Fig. 1).

### Study characteristics and quality evaluation

The characteristics of the included RCTs are presented in Table 1. Assessment of the study quality using the PEDro scale is shown in Table 2. The methodological quality of the included studies was good.

**Table 1:** Characteristics of the included RCTs

Study	Sample size (T/C)	Age (years)	Number of pregnancies (week)	Type of intervention		Duration of trial period	Outcomes
				T	C		
Sabbour 2011 [21]	30/30	29.08 ± 5.08	30.7 ± 4.96	KT + Exercise therapy+Low back pain health guidance	Exercise therapy+Low back pain health guidance	2 week Two applications each of continued for three days with one day as rest.	VAS, ODI
Şeyhmus 2016 [22]	33/32	24.30 ± 4.96/25.09 ± 4.95	21.79 ± 5.47/21.91 ± 3.86	KT + Paracetamol 50% tention	Paracetamol	5 days 5 days of additional Kinesio taping therapy.	VAS, RMDQ
Paweł 2017 [23]	53/53	29.5 ± 4.25	35.2 ± 4.3	KT 75% tention	Placebo taping	1 week 5 days of additional Kinesio taping therapy.	VAS, RMDQ
Matchimamart 2021 [24]	20/20	30.7 ± 4.0/30.2 ± 5.7	32.1 ± 3.4/33.3 ± 3.3	KT 50% tention	Placebo taping	1 week	VAS, RMDQ
FAN 2018 [25]	28/28	28.18 ± 3.95/28.00 ± 3.53	31.54 ± 2.82/32.64 ± 2.60	KT + Core strength training+Breathing training	Core strength training+Breathing training	2 week Two applications each of continued for three days with one day as rest.	VAS, RMDQ
YE J 2019 [26]	29/30	21 ~ 35	<16	KT + Exercise therapy+Low back pain health guidance 50% tention	Placebo taping+Exercise therapy+Low back pain health guidance	6 days Two times in total	VAS, ODI
YE M 2020 [27]	29/29	27.52 ± 2.97/27.43 ± 3.23	29.29 ± 0.45/29.24/0.25	KT + Psychosupportive therapy 20% tention	Psychosupportive therapy	4 week. Two applications each of continued for three days with one day as rest..	VAS, RMDQ

*T* Trail group, *C* Control group, *ODI* Oswestry Disability Index

**Table 2:** Assessment of the methodological quality using the PEDro scale

STUDY	PEDro Scale Items^a^											Total score (0–10)	Quality
	1	2	3	4	5	6	7	8	9	10	11		
Sabbour 2011	Y	Y	N	Y	N	N	N	Y	Y	Y	Y	7	Good
Şeyhmus 2016	Y	Y	Y	Y	Y	N	N	Y	Y	Y	Y	9	Excellent
Paweł 2017	Y	Y	Y	Y	Y	N	N	Y	Y	Y	Y	9	Excellent
Matchimamart 2021	Y	Y	Y	Y	Y	Y	N	Y	Y	Y	Y	10	Excellent
FAN 2018	Y	Y	N	Y	N	N	N	Y	Y	Y	Y	7	Good
YE J 2019	Y	Y	N	Y	N	N	N	Y	Y	Y	Y	7	Good
YE M 2020	Y	Y	N	Y	N	N	N	Y	Y	Y	Y	7	Good

Abbreviations: *N* no, *Y* yes

^a^PEDro Scale Items 1:eligibility criteria and source of participants; 2: random allocation; 3: concealed allocation; 4: baseline comparability; 5: blinded subjects; 6: blinded therapists; 7:blind assessors; 8: adequate follow-up; 9: intention-to-treat; 10:between-group comparisons; 11: point estimates and variability

### Results of Meta-analysis

#### About pain improvement: VAS scores

**Fig. 2:**
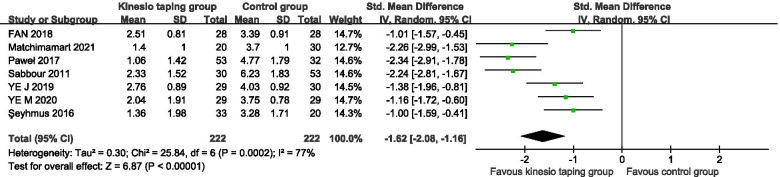
Forest plot of VAS pain improvement score

**Fig. 3:**
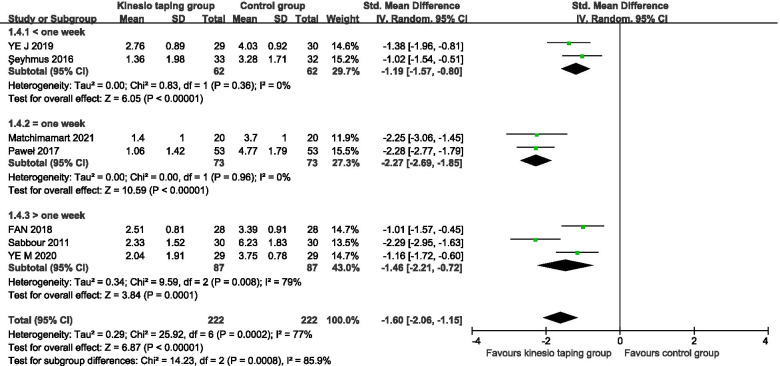
Forest plot of VAS pain improvement score subgroup for intervention cycle classification

**Fig. 4:**
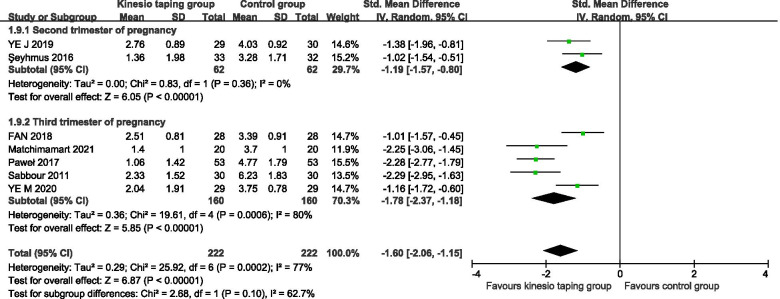
Forest plot of VAS pain improvement score subgroup of pregnancy cycle classification

A total of 7 RCTs were included [24–30], 444 pregnant women. The results of the meta-analysis of the random effects model showed that there was a statistically significant difference between the KT intervention group and the control group (− 1.62, 95%CI − 2.08 to − 1.16, *P* < 0.00001, I^2^ = 77%) (Fig. 2). The results of subgroup analysis showed that, according to the intervention cycle classification, compared with the control group, the KT group had less than 1 week of intervention, and the difference in the improvement of lumbar pain was statistically significant (− 1.19, 95%CI − 1.57 to − 0.80, *P* < 0.00001, I^2^ = 0%) (Fig. 3). Compared with the control group, the difference in the improvement of low back pain was statistically significant in the KT group after 1 week of intervention (− 2.27, 95%CI − 2.69 to − 1.85, *P* < 0.00001, I^2^ = 0%) (Fig. 3). Compared with the control group, the intervention in the KT group was more than 1 week, and the difference in the improvement of low back pain was statistically significant (SMD = -1.46, 95%CI − 2.21 to − 0.72, *P* = 0.0001, I^2^ = 79%) (Fig. 3). The heterogeneity was mainly derived from Sabbour’s study, (SMD = -1.08, 95%CI − 1.48 to − 0.69, *P* < 0.00001, I^2^ = 0%) [24]. According to the classification of pregnancy cycle, 14 to 28 weeks were divided into the second-trimester group, 28 to 40 weeks were divided into the third-trimester group. Compared with the control group, KT intervention had a statistically significant difference in the improvement of low back pain in the second and third trimesters of pregnancy (− 1.19, 95%CI − 1.57 to − 0.80, *P* < 0.00001, I^2^ = 0%), (SMD = -1.78, 95%CI − 2.37 to − 1.18, *P* < 0.00001, I^2^ = 80%) (Fig. 4).

#### About dysfunction improvement: RMDQ scores

**Fig. 5:**
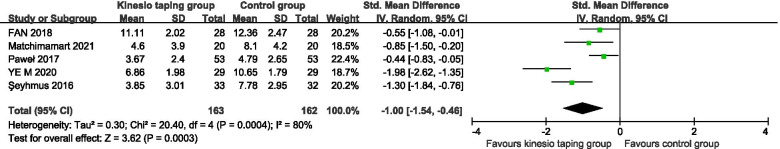
Forest plot of RMDQ dysfunction improvement score

**Fig. 6:**
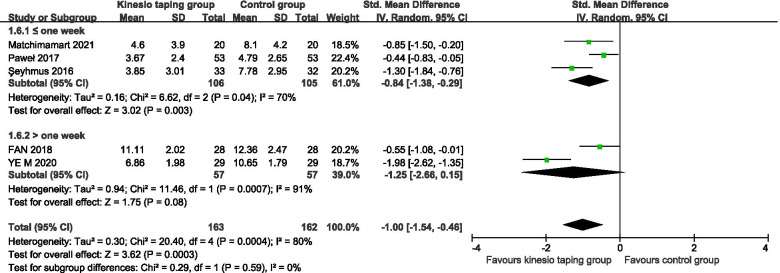
Forest plot of RMDQ dysfunction improvement score subgroup of the intervention cycle classification

A total of 7 RCTs were included [25–28, 30],444 pregnant women. The results of the meta-analysis of the random effects model showed that there was a statistically significant difference between the KT intervention group and the control group (− 1.00, 95%CI − 1.54 to − 0.46, *P* = 0.0003, I^2^ = 80%) (Fig. 5). The results of subgroup analysis showed that, according to the intervention cycle classification, compared with the control group, the KT group was treated for less than or equal to 1 week, and the improvement difference of lumbar dysfunction was statistically significant (− 0.84, 95%CI − 1.38 to − 0.29, *P* = 0.003, I^2^ = 70%) (Fig. 6); Compared with the control group, the intervention in the KT group was more than 1 week, and there was no statistically significant difference in the improvement of lumbar dysfunction (− 1.25, 95%CI − 2.66 to 0.15, *P* = 0.08, I^2^ = 91%) (Fig. 6).

## Discussion

This is the first systematic review and meta-analysis designed to evaluate the efficacy and safety of KT in low back pain during pregnancy, involving seven clinical trials. The results of this study showed that, compared with the control group, KT intervention could significantly improve the low back pain and lumbar dysfunction, and the VAS score and RMDQ score showed statistically significant differences. The results of the subgroup meta-analysis showed that compared with the control group, the low back pain and lumbar dysfunction were improved after KT intervention for less than or equal to 1 week, with statistically significant differences. After intramuscular effect patch intervention for more than 1 week, there was a statistically significant difference in the improvement of low back pain, but the RMDQ score showed no statistically significant difference. Compared with the control group, the intervention of KT improved the low back pain in the second and third trimesters, and the differences were statistically significant. This suggests that KT has a positive effect on the improvement of low back pain and lumbar dysfunction during pregnancy, superior to other methods, including placebos, drugs, and other general physical therapy methods, and can therefore serve as a useful therapeutic alternative. Based on the results of our systematic review, more studies should be conducted aimed at evaluating the medium and long-term effects of KT, and further studies on the long-term effects of KT on low back pain during pregnancy are needed. In addition, the duration of KT use should be further studied, as there is no consensus on the optimal dose for this intervention.

Pregnancy low back pain is often considered to be a natural painful process during pregnancy and is a common condition during pregnancy [31, 32]. It usually occurs between the fifth and seventh months of pregnancy, and in some cases, pregnancy pain in the lower back can begin as early as eight to 12 weeks into the pregnancy. Women who have lower back problems before pregnancy are at higher risk for lower back pain, which can start early in pregnancy [33, 34]. A follow-up survey of 1131 pregnant women in the United States found that 0.4% of low back pain occurred in early pregnancy, 24.4% in the second trimester, and 75.1% in the third trimester. The incidence of low back pain increased with the increase of gestational weeks and reached a peak at 36 weeks of gestation, and with the increase of gestational cycles, the pain would gradually worsen [35, 36]. Therefore, early or early intervention is timely and necessary for the improvement of low back pain during pregnancy. The common treatment methods of low back pain during pregnancy include multimodal intervention (manual therapy, exercise, and health education), exercise therapy, and acupuncture. Among them, various forms of exercise therapy are the most commonly used intervention. However, a recent systematic review reported that the effect of exercise improvement is relatively low, and the improvement effect of acupuncture and intramuscular plaster is obvious [31, 37]. Since patients and clinicians tend to avoid drugs and invasive treatment during pregnancy, KT as a drug-free and safe alternative therapy provides a new treatment for low back pain during pregnancy. The improvement effect of functional movement is better than physical therapy, and it will not affect the fetus. It can minimize the occurrence and development of chronic pain and is simple, convenient, noninvasive, painless, and radiation-free. The mechanism of KT may not be completely clear, but as physical therapy, KT is widely used in the clinic. In recent years, clinical attention has been paid to the effect of KT on low back pain during pregnancy, and relevant studies have been published, showing that KT has a significant effect on low back pain during pregnancy [27, 34].

To compare the treatment effect of KT on pregnant women’s low back pain during pregnancy, this study selected the low back pain scoring scale commonly used in clinical practice, which can evaluate the degree of low back pain and lumbar injury, select the treatment plan and evaluate the treatment effect. At present, the scoring criteria commonly used in the world for lumbar dysfunction include Roland Morris Dysfunction Questionnaire (RMDQ), Oswestry Disability Index (ODI), Quebec Back Pain Disability Scale (QBPDS), JOA Low Back Pain Assessment Scale, Visual Analogue Scale (VAS), etc. Among them, the visual analogue scale of pain and Roland Morris dysfunction questionnaire are widely used as clinical outcome indicators in the study of the intervention of low back pain with KT during pregnancy, and they are also the most commonly used scale for the evaluation of low back pain in the world. The Roland-Morris Dysfunction Inventory was created by Roland and Morris in 1983. It can evaluate short-term changes before and after treatment of low back pain. It is a specific scale to evaluate lumbar dysfunction, with a minimum score of 0 and a maximum score of 24 [38–40]. Therefore, the visual analogue scale of pain and the Roland Morris Dysfunction Inventory was selected as clinical outcome indicators.

Careful assessment of pregnant women’s low back pain during pregnancy, the clear purpose of treatment, accurate identification of target muscle sticking, these factors have an important impact on the success of treatment. In the included studies, 3 studies [26, 27, 29] used KT versus placebo patches, which proved that the application of KT alone had a positive effect on reducing pregnant women’s low back pain and improving physical function during pregnancy; 2 studies [24, 28] used KT combined with routine rehabilitation training and routine rehabilitation training to confirm that KT can significantly improve the posture, pain and function of patients with low back pain during pregnancy; One study [25] used KT combined with analgesics, and the results showed that compared with paracetamol alone, KT combined with paracetamol effectively reduced pain and improved function, indicating that KT can be used as an adjuvant treatment to achieve effective control of low back pain during pregnancy. One study [30] adopted KT combined with psychotherapy, which effectively alleviated low back pain, anxiety, and depression of pregnant women during pregnancy.

At present, the pathophysiological mechanisms associated with low back pain during pregnancy are not clear. The main agreed factors are: First, weight gain, postural changes, and hormonal fluctuations during pregnancy, it may cause problems in the musculoskeletal system, destabilizing the spine and sacroiliac joint as well as connective tissue. Second, relaxation hormone, as a hormone secreted by the placenta, relaxes pelvic ligaments and the ligaments that support the spine, especially in late pregnancy, which may cause lower back pain [41]. In addition, pregnant women are generally less engaged in physical labor and sports, and more sedentary work, the lumbar and back muscles can’t be effectively exercised, and the core muscle strength is weak, which is also one of the reasons for low back pain during pregnancy. Aleksandra et al., based on a study of 1510 pregnant women, found that the main risk factors for low back pain during pregnancy were onset of low back pain before pregnancy or menstruation, younger age, and lack of physical activity [4]. Therefore, the main goal of treatment is to reduce the intensity of pain, restore function and prevent the pain from becoming chronic. For pregnant women with low back pain, there is a tendency to avoid medication for fear of side effects and a preference for non-invasive and non-invasive treatment. Treatments focus on maintaining proper postures, movement adjustments, and health education.

The results of the current study show that the KT intervention significantly improved low back pain and dysfunction in pregnant women compared to other treatments in the control group, which is consistent with the results of previous studies [17, 27]. Possible mechanisms by which intramuscular tape improves low back pain during pregnancy include improved lower-back stability and increased proprioception, which in turn improves postural control [42, 43]; In addition, the KT can effectively adhere to the skin and exert pressure, increase the space under the skin or between the dermis and epidermis, promote subcutaneous blood circulation and lymphatic return, and accelerate the healing of the injured site through its tension, thus helping to eliminate substances that cause pain; KT also provides a continuous neurosensory input to the skin receptors, thereby relatively suppressing the sensory input of pain and improving their ability to reduce mechanical stimulation of soft tissue during lumbar spine movement [44, 45]. Pain relief is the most important criterion in treatment because pain can seriously affect a pregnant woman’s daily life. The key to using KT to relieve pain is how to choose the appropriate location, adjust the appropriate tension and determine the time of adhesive. Senbursa’s study found that KT was very effective in improving low back pain in a short period, and could immediately show pain relief during activity and relaxation without other adverse reactions [46]. However, some studies have found that the KT can significantly improve the pain, range of motion, and injury of patients with low back pain in the short term, and maintain the improvement of range of motion and injury in the long term, but there is no long-term effect on the improvement of pain [47]. Therefore, the long-term effects of KT on low back pain during pregnancy still need to be further studied. In addition, in the United Kingdom and the United States, treatment of low back pain during pregnancy usually includes health education on low back pain, postural and body mechanics education starting in the first trimester, such as the type of pillow to sleep on, and physical therapy [10]. Education and guidance for pregnant women, popularize the knowledge of health care related to low back pain, to reduce the occurrence of low back pain during pregnancy, which is also the content of health education for pregnant women by obstetrics and gynecology and related medical personnel.

This meta-analysis study suggested that after KT intervention for low back pain during pregnancy, the improvement degree of low back pain and dysfunction in the experimental group was better than that in the control group, and the differences were statistically significant, suggesting that intramuscular effect patch intervention had a good effect on the improvement of low back pain and dysfunction during pregnancy. The results of the subgroup analysis showed that, according to the classification of pregnancy cycle, compared with the control group, KT intervention had statistically significant differences in the improvement of waist pain in the second and third trimesters of pregnancy. According to the classification of intervention cycle, compared with the control group, there were statistically significant differences in the improvement of lumbar pain in the KT group after intervention for 1 week, less than 1 week, and greater than 1 week. In the KT group, the intervention was less than or equal to 1 week, and the improvement difference of lumbar dysfunction was statistically significant. However, when the intramuscular effect patch group was treated for more than 1 week, there was no statistically significant difference in the improvement of lumbar function, and there was great heterogeneity. The analysis reasons might be as follows: Subgroup analysis indicated that the effect of intramuscular adhesive was different with different intervention time and pregnancy cycle, which may be the source of heterogeneity. There was no gold standard for the application of KT and there were differences among treatment regimens, which might lead to different therapeutic effects; The measurement of outcome indicators is affected by subjective factors, which leads to the deviation of data; There is no unified standard of routine rehabilitation training, and its intervention measures are inconsistent, which may also lead to heterogeneity; The specific measures taken by different control groups were not completely the same, which may be the source of heterogeneity.

One similar review has been conducted on this topic, a meta-analysis of RCTs by Chen et al. [48] has also reported almost similar findings compared to our review. However, the Systematic Review and Network meta-analysis included two studies on the effects of KT intervention on pregnant women’s low back pain. Other studies included were physiotherapy or drug therapy, and the intervention methods and time of the control group were very heterogeneous. Besides, the title of one [49] of the studies is “Efficiency of kinesiotape on functional disability of women with postnatal back pain: A randomized controlled trial”, the main discussion is about the improvement effect of KT on low back pain after delivery, while our study aims to discuss the effect of KT on low back pain during pregnancy. We believe that the latest evidence will help healthcare personnel to correctly choose the sticking method and time of low back pain during pregnancy.

## Strength and limitations

A strength of the present study is that includes the comprehensive search of the literature and the broad search strategy resulting in the inclusion of all studies published on the topic to date. The data reporting adheres to the Preferred Reporting Items for Systematic reviews and Meta-Analyses guidelines. A large scope of evidence was available and retrieved from the Cochrane Library, Web of Science, and Embase. We only included RCTs into our review which enables us to infer causal associations between the intervention and outcomes. The outcome was analyzed according to the severity of low back pain and lumbar dysfunction score, so as to fully consider the impact of these factors on the overall outcome. Although this study resulted in some meaningful implications, it also has some limitations. There are some limitations in this study: The relatively small number of included studies and sample size affect the reliability of the conclusions; Most of the included studies did not report allocation concealment or blind method, which has a certain risk of bias; Visual analogue scale of VAS pain is greatly influenced by subjective factors, which may lead to data deviation among different studies; There are differences in the shape, sticking method, location, and length of intramuscular effect stickers in different studies, which may also lead to clinical heterogeneity; The heterogeneity for certain comparations was significant, which may have influenced the pooled results, despite our using a random-effects model. Two subgroups analyses were performed according to intervention time, classification of pregnancy cycle, but other factors that could influence the outcomes might be present.

## Conclusion

This meta-analysis demonstrated that KT, either separately or in combination with other general therapies, resulted in greater pain relief and improvement in RMDQ in patients with PLBP than did general physical therapies without KT. It is suggested that future research should focus on the prevention and treatment of low back pain during pregnancy to provide more research data for improving women’s health.

## Supplementary Information

**Additional file 1.** S1 Checklist. PRISMA checklist.

**Additional file 2.** S1 File. Search strategy for the PubMed database.

## Data Availability

The data sets analyzed during the current study will be available upon reasonable request of the corresponding author.
