# Supplementary material for: Effect of kinesio taping on low back pain during pregnancy: a systematic review and meta-analysis
Source: BMC Pregnancy Childbirth. 2021 Oct 25;21:712. doi: 10.1186/s12884-021-04197-3 (PMC8547085; doi:10.1186/s12884-021-04197-3)
Supplement: Supplementary file 2 — Additional file 2. S1 File. Search strategy for the PubMed database. [file 12884_2021_4197_MOESM2_ESM.docx]

**Search strategy for the PubMed database.**

| **Number** | **Search items** |
| --- | --- |
| 1 | Kinesiotape |
| 2 | Kinesio Tape |
| 3 | Kinesio Tapes |
| 4 | Tape, Kinesio |
| 5 | Tapes, Kinesio |
| 6 | Athletic Tape |
| 7 | Tape, Athletic |
| 8 | Orthotic Tape |
| 9 | Tape, Orthotic |
| 10 | 1 or 2- 9 |
| 11 | Pregnancy |
| 12 | Pregnancies |
| 13 | Gestation |
| 14 | 11 or 12-13 |
| 15 | randomized controlled trial |
| 16 | randomized |
| 17 | clinical trial |
| 18 | randomly |
| 19 | Controlled clinical trials |
| 20 | controlled before-after studies |
| 21 | 15 or 16-20 |
| 22 | 10 and 14 and 21 |
